# Dr. William Q. Huggins

**Published:** 1912-11

**Authors:** 


Dr. William Q. Huggins of Sanborn died Oct. 21, 1912. He was born at Roanoke, Devonshire, Eng., but came to the U. S. when a small boy. He received the degree of M. D. from a Cincinnati college in 1861, enlisted Battery E, 1st N. Y. Artillery and served with honor through the Civil War, being wounded several times in battle of Lookout Mountain and being captured during Sherman's march to the sea. He escaped and re-entered active service, being discharged in April, 1865. In 1870, he graduated in medicine from the University of Medicine, after having spent a couple of years in California recuperating his health. After his second graduation in medicine, he located in Sanborn, where he has since resided and practiced his profession. He was a Mason, an Odd Fellow and a member of various other organizations, including the G. A. R. From his gallant military service, we may gain an idea of his character.

